# Correlation Among Psoriasis, Iridocyclitis, and Non-alcoholic Fatty Liver Disease: Insights from Mendelian Randomization and Mediation Analysis

**DOI:** 10.7150/ijms.102369

**Published:** 2025-01-01

**Authors:** Shuqin Xu, Long Liu, Chentao Li, Yaoxing Ren, Miaomiao Zhang, Linbiao Xiang, Nan Li, Jiaru Xu, Shuang Bai, Yi Lv

**Affiliations:** 1 Department of hepatobiliary surgery, the First Affiliated Hospital of Xi'an Jiaotong University, Xi'an 710061, China.; 2 National Local Joint Engineering Research Center for Precision Surgery & Regenerative Medicine, the First Affiliated Hospital of Xi'an Jiaotong University, Xi'an 710061, China.; 3 Bioscience and Biomedical Engineering Thrust, The Hong Kong University of Science and Technology (Guangzhou), Guangzhou 511457, China.; 4 School of Future Technology, Xi'an Jiaotong University, Xi'an 710061, China.; 5 Shaanxi Provincial Key Laboratory of Magnetic Medicine, the First Affiliated Hospital of Xi'an Jiaotong University, Xi'an 710061, China.; 6 Department of Ophthalmology, the First Affiliated Hospital of Xi'an Jiaotong University, Xi'an 710061, China.; 7 Zonglian College, Xi'an Jiaotong University, Xi'an 710061, China.; 8 Shaanxi Province Center for Regenerative Medicine and Surgery Engineering Research, the First Affiliated Hospital of Xi'an Jiaotong University, Xi'an 710061, China.

**Keywords:** Psoriasis, Iridocyclitis, NAFLD, Mendelian randomization, Mediation effect

## Abstract

**Purpose:** The aim of this study is to utilize two-sample Mendelian randomization (MR) to investigate the potential causal relationship among psoriasis, iridocyclitis, and non-alcoholic fatty liver disease (NAFLD), and to explore any potential mediation effects.

**Methods:** Pooled data were derived from the public genome-wide association study (GWAS) in NAFLD (finn-b-NAFLD), iridocyclitis (finn-b-H7_IRIDOCYCLITIS) and psoriasis (finn-b-L12_PSORI_VULG). Univariable MR (UVMR) analysis was implemented to explore the causal relationship among psoriasis, iridocyclitis, and NAFLD, and inverse variance weighting (IVW) was used as the primary analytical method. Additionally, Cochran's Q and MR-Egger tests were utilized to evaluate the heterogeneity and horizontal pleiotropy, respectively. Simultaneously, the reliability of MR results was evaluated by leave-one-out (LOO) method. Finally, multivariable MR (MVMR) analysis and mediation analysis were performed to further reveal the mechanism of mediation effect among the three diseases.

**Results:** With regard to the results of IVW method, both iridocyclitis (*P*=0.0185, OR=1.0757) and psoriasis (*P*=0.0115, OR=1.1246) had significant causal relationships with the occurrence of NAFLD, and both were risk factors for NAFLD. Besides, it was observed that there was significant causal effect of iridocyclitis (*P*= 0.0181, OR=1.1729) on psoriasis and iridocyclitis was a risk factor. Additionally, there was a lack of heterogeneity (*P*>0.05) among the selected SNPs when MR analysis was conducted with NAFLD as the outcome. Horizontal pleiotropy was not detected by the MR-Egger test. The LOO analysis demonstrated that the instrumental variables were appropriately chosen, suggesting the reliability of the MR results. Ultimately, MVMR and mediation analysis revealed iridocyclitis affected the development of NAFLD, 20.81% of which was caused by the pathway of iridocyclitis induced psoriasis leading to NAFLD.

**Conclusion:** This study highlighted that iridocyclitis was significantly associated with an increased risk of NAFLD and that psoriasis was involved in the mechanism by which iridocyclitis triggered NAFLD, which might offer potential preventive strategies for NAFLD.

## Introduction

Non-alcoholic Steatohepatitis (NASH) was initially conceptualized by Ludwig et al. 1 in 1980. Non-alcoholic fatty liver disease (NAFLD) is defined as the presence of hepatic steatosis in more than 5% of hepatocytes, coupled with metabolic risk factors, and without significant alcohol consumption (≥30 g/day for men, ≥20 g/day for women), or other chronic liver diseases 2. Due to its strong association with metabolic syndrome, NAFLD is prevalent among patients with type 2 diabetes mellitus and obesity 3, 4. Currently, NAFLD stands as the leading etiology of chronic liver disease worldwide, estimated to affect approximately 25% of the global population, with over 80 million individuals affected in the United States alone 5. The prevalence of NAFLD varies geographically, ranging from 13.5% in Africa to 31.8% in the Middle East. These regional variations may be attributed to differences in dietary patterns 5, adipose tissue distribution, socioeconomic status, and genetic composition. With global aging trends and the rising incidence of diabetes and obesity, the incidence of NAFLD is projected to escalate significantly over time 6. Despite only 10% of NAFLD patients develop complications such as cirrhosis or hepatocellular carcinoma within 20 years of diagnosis, the high prevalence of NAFLD imposes a substantial disease and economic burden that is likely to intensify in the coming decades 7. Therefore, exploring the pathogenesis of NAFLD is imperative. Nutrient excess serves as the primary driving factor of NAFLD, leading to ectopic fat accumulation. In this milieu, macrophage infiltration into adipose tissue promotes a pro-inflammatory state, further exacerbating insulin resistance. Under conditions of insulin resistance, inappropriate fatty acid influx to the liver increases, coupled with* de novo* lipogenesis, exacerbating hepatic metabolic burden 8. However, the pathogenic pathways of NAFLD are influenced by multiple metabolic, genetic, and microbial factors, which remain incompletely elucidated, and definitive causal relationships have yet to be established 8. Genetic risk variants demonstrate a synergistic interplay between NAFLD and obesity 9.

Psoriasis, an immune-mediated genetic disorder, may manifest in the skin, joints, or both, with males often experiencing more severe symptoms than females 10, 11. Characterized by dysregulated interactions between innate and adaptive immunity with the skin cell population, psoriatic skin lesions ensue 10. Iridocyclitis, a form of uveitis, initiates its inflammation in the anterior portion of the uvea, specifically the iris and ciliary body, posing a common inflammatory eye ailment that can potentially impair vision 12, 13. Predominantly affecting children and young adults, it frequently coexists with systemic diseases such as ankylosing spondylitis, reactive arthritis, and other spondyloarthropathies 14, 15, with nearly 50% of acute iridocyclitis patients testing positive for human leukocyte antigen B27 (HLA-B27) 14. A recent cohort study revealed that newly diagnosed psoriasis patients are 1.28 times more likely to develop NAFLD in the future compared to their non-psoriatic counterparts 16. Although limited studies have elucidated the clinical correlations between psoriasis, NAFLD, and iridocyclitis, the specific relationships between psoriasis and iridocyclitis remain inadequately elucidated. Nevertheless, recent reports indicate an increasing prevalence of concurrent psoriasis and uveitis 17-19.

Mendelian randomization (MR) analysis, an epidemiological study design employing single nucleotide polymorphisms (SNPs) as instrumental variables (IVs), elucidates the causal impact of exposure on outcomes. This approach represents a natural randomized controlled trial, relying on the random allocation of alleles during gamete formation. Compared to traditional observational multivariable regression, MR minimizes confounding and reverse causality. Bidirectional MR analysis, an extension of conventional MR, disentangles complex relationships within biological systems, including feedback loops between exposure factors and outcome variables. Essentially, it evaluates whether reverse causality exists between exposure and outcome, i.e., whether the outcome influences exposure occurrence 20, 21. Although existing studies have substantiated clinical correlations among psoriasis, NAFLD, and iridocyclitis, deeper mechanistic understanding and causal relationships among these diseases, particularly lacking genetic evidence, necessitate the utilization of SNPs data and bidirectional MR analysis.

This study employs SNPs data from public databases on iridocyclitis, psoriasis, and NAFLD to investigate causal relationships among the three diseases using two-sample MR method. Sensitivity analyses were conducted to assess the impact of assumptions on investigative outcomes and ensure result robustness. Furthermore, mediation analysis was performed to furnish novel genetic evidence for the causal relationships among iridocyclitis, psoriasis, and NAFLD from a genetic perspective.

## Materials and methods

### Study design

This study employed a bidirectional and two-sample MR design to examine the reciprocal causal association among psoriasis, iridocyclitis, and NAFLD. The MR design was based on three fundamental assumptions: (1) Correlation assumption, IVs exhibited significant correlations with exposure factor; (2) Independence assumption, IVs were independent of confounding factors; and (3) Exclusivity hypothesis, IVs could solely influence the outcome through exposure factor. Pooled statistics derived from publicly available genome-wide association studies (GWAS) were utilized for analysis purposes, eliminating the need for additional ethical approval.

### Data source

All data extraction for this study was conducted using the Integrated Epidemiology Unit (IEU) Open GWAS database (https://gwas.mrcieu.ac.uk/), and individuals of European descent formed the foundation for this investigation. Specifically, the NAFLD-related summary data (finn-b-NAFLD) included 16,380,466 SNPs from 218,792 samples (894 NAFLD and 217,898 control samples), the iridocyclitis-related summary data (finn-b-H7_IRIDOCYCLITIS) included 16,380,395 SNPs from 212,909 samples (3,622 iridocyclitis and 209,287 control samples), and the psoriasis-related summary data (finn-b-L12_PSORI_VULG) included 16,380,459 SNPs from 215,044 samples (2,802 psoriasis and 212,242 control samples).

### Selection of IVs

To ensure the selection of qualified instrumental variables (IVs), this study implemented several quality control steps. Firstly, SNPs significantly correlated with exposure factor were selected as IVs from GWAS pooled data utilizing extract_instruments function from R package-'TwoSampleMR' (v 0.5.6) 22, with *P*<5×10^-8^ as the threshold (psoriasis and iridocyclitis as exposure factors), In particular, in the case of a limited number of IVs, the significance threshold was relaxed to 5×10^-6^ (NAFLD as exposure factor) to prevent inaccurate results due to insufficient SNPs. Following this, linkage disequilibrium (LD) clumping was utilized to exclude some unwanted SNPs (r^2^<0.001, window size>10,000 kb) with the aim of ensuring SNPs independence. Eventually, IVs that exhibited significant association with the outcome were excluded, and the 'harmonise_data' function from R package-'TwoSampleMR' was utilized to harmonize the effect alleles and effect sizes. Additionally, calculating the bias introduced by Mendelian sample overlap through the online website at https://sb452.shinyapps.io/overlap/.

### Univariable MR (UVMR) analysis and sensitivity analysis

We performed a univariable MR analysis to explore the causal relationship among psoriasis, iridocyclitis, and NAFLD. In other words, six MR analyses were implemented with each of the three diseases as outcomes and the other two diseases as exposure factors. Five different approaches, including MR Egger 23, which detected and corrected for bias due to weak instrumental variables. Weighted median 24, which could more robust in dealing with some cases where the instrumental variables were weak or heterogeneity exists. Inverse variance weighted (IVW) 25, it provided maximum statistical efficacy with multiple valid instrumental variables. Simple mode did not take into account precision or heterogeneity of instrumental variables, and weighted mode 26, It took into account the frequency and precision of effect sizes 27, were employed to establish the presence of statistically significant causality. Among them, IVW was considered to be the primary analytical method, which played a decisive role in judging the causal effect. A significance level of *P*<0.05 indicated a notable causal effect of exposure factor on outcome. Additionally, odds ratio (OR) value was computed where OR>1 meant that exposure factor acted as a risk factor, while OR<1 meant its role as a protective factor.

To enhance the reliability of the MR analysis results, sensitivity analysis was carried out. Initially, Cochran's Q-test was employed to assess heterogeneity, with a *P*-value greater than 0.05 indicating a lack of significant heterogeneity. Immediately after that, potential horizontal pleiotropy among IVs was evaluated using MR-Egger test, and a *P*-value exceeding 0.05 suggested the absence of horizontal pleiotropy. Additionally, leave-one-out (LOO) analysis was carried out to check whether MR analysis results were affected by any SNPs and further validate the robustness of the results.

### Multivariable MR (MVMR) analysis and mediation analysis

The results of UVMR analysis were labeled in the mediation effect analysis diagram to infer the possible mediation effect direction. In order to further explore the mechanism of the mediation effect, the corresponding exposure factor and outcome were selected to design MVMR analysis. IVs screening conditions in MVMR analysis were as follows: (1) SNPs strongly associated with the exposure factors were identified at a genome-wide significance level (*P* < 5×10^-6^); (2) A criterion (r^2^<0.001, kb=10,000) was applied to select SNPs that were independent of LD; (3) The final IVs were screened through reading the outcome data and combining with IVs in the exposure factors (proxies=TRUE, rsq=0.8).

Further, based on the causal relationship among psoriasis, iridocyclitis, and NAFLD in UVMR and MVMR analyses results, the exposure factor, mediation factor and outcome were correspondingly selected for implementing mediation analysis as well as calculating the mediation effect size and proportion. The calculation formulas were below: (1) 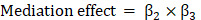; (2) 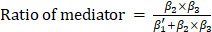.

## Results

### Causal relationships among psoriasis, iridocyclitis, and NAFLD

**Figure 1:**
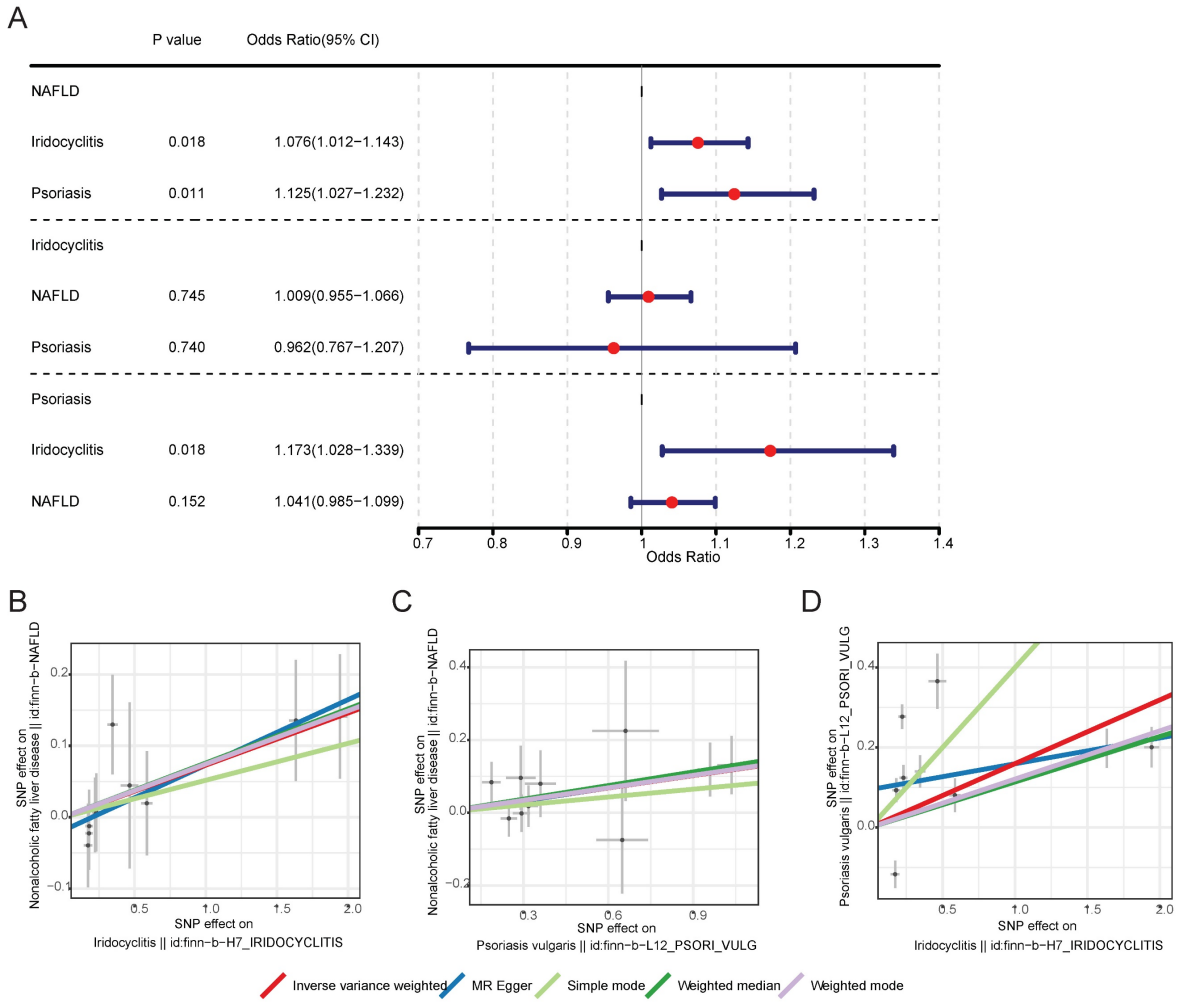
MR results and scatter plots of causal estimates for psoriasis, iridocyclitis, and NAFLD. (A) MR results of causal estimates for psoriasis, iridocyclitis, and NAFLD. Scatter plots of causal estimates for (B) NAFLD on iridocyclitis; (C) NAFLD on psoriasis; (D) psoriasis on iridocyclitis. MR, Mendelian randomization; NAFLD, Non-alcoholic fatty liver disease; OR, odds ratio; SNP, single nucleotide polymorphism.

After screening, 10 IVs for iridocyclitis and 9 IVs for psoriasis were obtained in MR analysis of NAFLD as outcome, 12 IVs for NAFLD and 9 IVs for psoriasis were obtained in MR analysis of iridocyclitis as outcome, as well as 12 IVs for NAFLD and 10 IVs for iridocyclitis were obtained in MR analysis of psoriasis as outcome. **Supplementary Table 1** demonstrated the UVMR results of five different methods. With regard to the results of IVW method (**Table 1** and** Fig. 1)**, both iridocyclitis (*P*=0.0185, OR=1.0757) and psoriasis (*P*=0.0115, OR=1.1246) had significant causal relationships with the occurrence of NAFLD, and both were risk factors for NAFLD. There was no significant causal relationship of psoriasis and iridocyclitis on NAFLD (*P*>0.05). Besides, it was observed that there was significant causal effect of iridocyclitis (*P*= 0.0181, OR=1.1729) on psoriasis and iridocyclitis was a risk factor, but NAFLD had no significant causal relationship with psoriasis (*P*>0.05).

**Figure 2:**
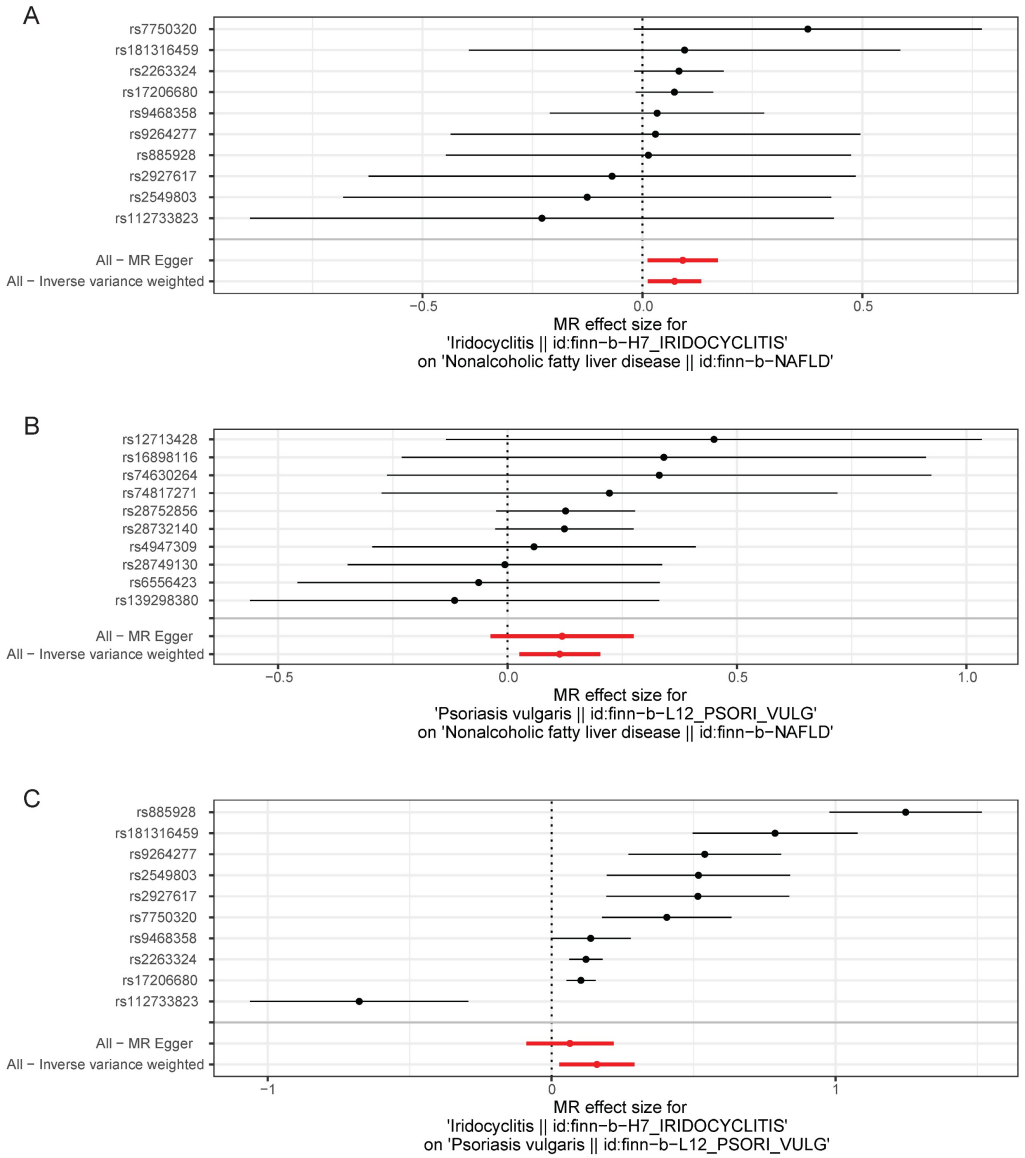
Forest plots of causal effect estimates of the genetic effects of SNPs in (A) iridocyclitis on NAFLD; (B) psoriasis on NAFLD; (C) iridocyclitis on psoriasis. Estimates were derived via MR using the IVW method. IVW, inverse variance weighting; MR, Mendelian randomization; NAFLD, Non-alcoholic fatty liver disease.

**Figure 3:**
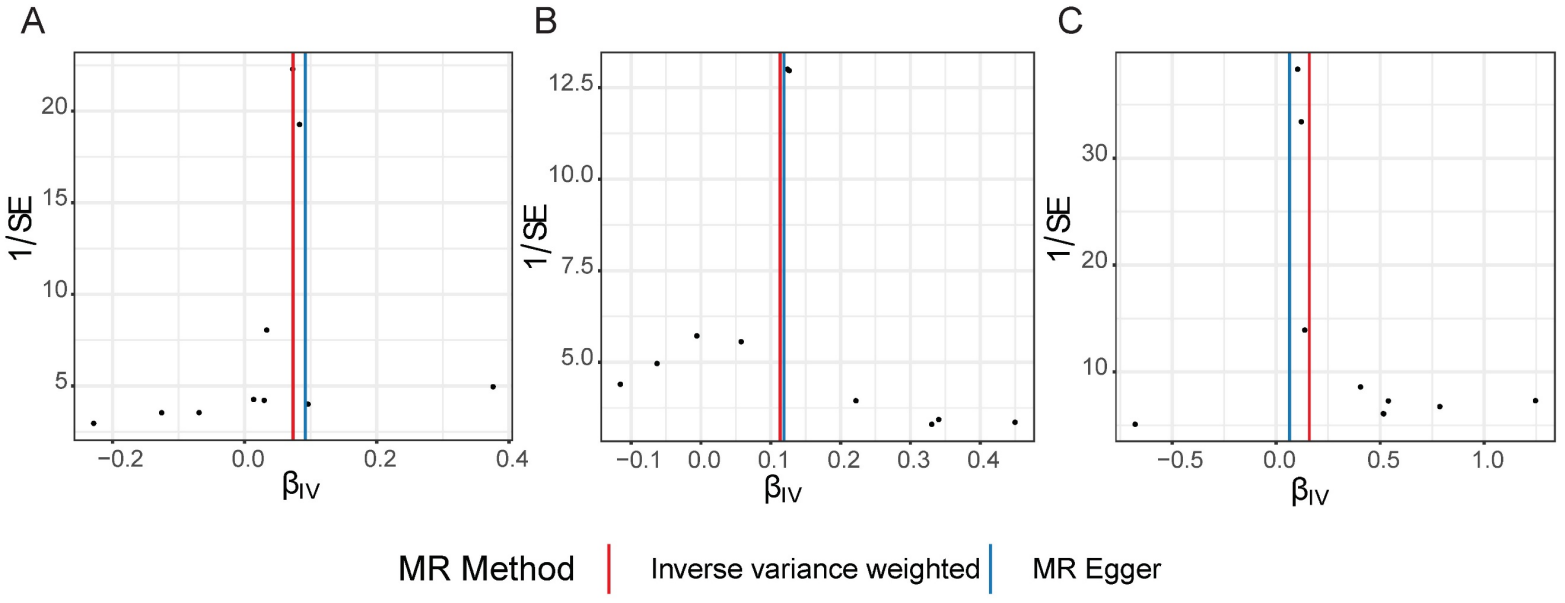
Funnel plots of causal effect estimates for A on B, with all X valid instrumental variables. (A) iridocyclitis on NAFLD; (B) psoriasis on NAFLD; (C) iridocyclitis on psoriasis. Estimates were derived via MR using the IVW method. IVW, inverse variance weighting; MR, Mendelian randomization; NAFLD, Non-alcoholic fatty liver disease.

Scatter plots, funnel plots and forest plots were demonstrated with the aim of having a more intuitive understanding of these significant causal relationships. The positive slope of the lines in scatter plots and the significant MR effect sizes greater than 0 in forest plots provided further support for the notion that both iridocyclitis and psoriasis were risk factors for NAFLD as well as iridocyclitis was a risk factor for psoriasis (**Fig. 1-2**). Simultaneously, the funnel plots indicated that MR analysis adhered to Mendel's second law of random grouping, suggesting that the study results were in line with the expected patterns of genetic inheritance and randomization (**Fig. 3**).

### UVMR analysis results were proven to be reliable through sensitivity analysis

**Figure 4:**
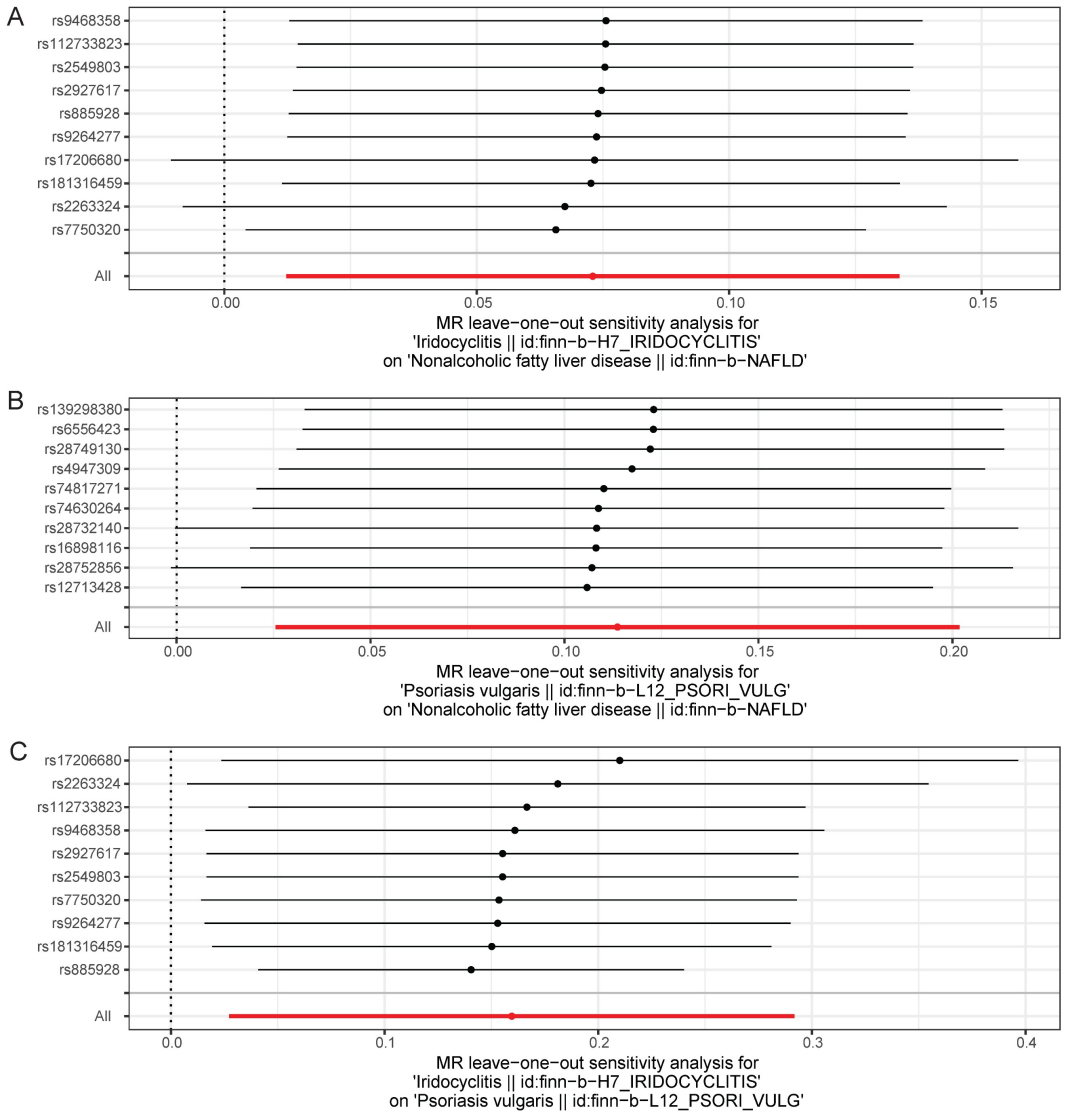
Leave-one-out test plots of causal effect estimates by UVMR for (A) iridocyclitis on NAFLD; (B) psoriasis on NAFLD; (C) iridocyclitis on psoriasis. UVMR, Univariable MR; NAFLD, Non-alcoholic fatty liver disease.

With regard to Cochran's Q-test, there was a lack of heterogeneity (*P*>0.05) among the selected SNPs when MR analysis was conducted with NAFLD as the outcome, and there was heterogeneity (*P*<0.05) in the causal effect of iridocyclitis on psoriasis (**Table 2**). Despite the presence of heterogeneity, the IVW method was used for analysis in this study, and a significant causal relationship was obtained. Meanwhile, the results of MR-Egger test provided evidence of a lack of horizontal pleiotropy among IVs (*P*>0.05) (**Table 3**). Moreover, the LOO analysis revealed that the exclusion of any individual SNP did not significantly impact the remaining SNPs results, affirming the reliability and robustness of the UVMR analysis results (**Fig. 4**). Additionally, we calculated the results for each SNP and all SNPs had F-values greater than 10, and also included consideration of the coefficient of determination, R^2^, to measure the explanatory power of the instrumental variables, with all R^2^ thresholds set at 0.001, suggesting that the assignment of SNPs was completely randomized (**Supplementary Table 2-7**). We further calculated the sample overlap rate, from **Table 4-5**, it could be seen that when the overlap rate was 1, the error rate remained the same as when it was 0, which had no effect on the results.

### Iridocyclitis could affect NAFLD through psoriasis

**Figure 5:**
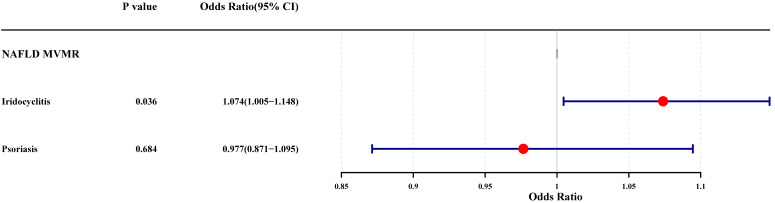
Forest plot of the MVMR analysis for iridocyclitis and psoriasis on NAFLD. MVMR, Multivariable MR; NAFLD, Non-alcoholic fatty liver disease.

The results of UVMR analysis were labeled in the mediation effect analysis diagram, and it was noted that only one mediation effect was valid, that is, iridocyclitis could lead to the occurrence of NAFLD by influencing the occurrence of psoriasis (**Supplementary Fig. 1**). Subsequently, the MVMR analysis was carried out with iridocyclitis and psoriasis as exposure factors and NAFLD as outcome, to explore the causal relationship. After the screening process, 6 SNPs associated with iridocyclitis and 5 SNPs related to psoriasis were selected as IVs. The results of the MVMR analysis were demonstrated in **Fig. 5**, where the significant causal effects of iridocyclitis on NAFLD (*P*=0.0362, OR=1.0739) were found, and iridocyclitis was still risk factors. However, the causal effect of psoriasis on NAFLD became insignificant.

According to the results of UVMR and MVMR analysis, we determined iridocyclitis as the exposure factor, psoriasis as the mediation factor, and NAFLD as the outcome to complete the mediation analysis. The results indicated that the direct effect value was 0.0713, mediation effect value was 0.0187, and the proportion of the mediator was 20.81% (**Table 6**). In other words, iridocyclitis affected the development of NAFLD, 20.81% of which was caused by the pathway of iridocyclitis induced psoriasis leading to NAFLD.

## Discussion

In this study, we utilized MR analysis to investigate the causal relationships among iridocyclitis, psoriasis, and NAFLD. In our univariable MR analysis, we found that both iridocyclitis and psoriasis were associated with an increased risk of NAFLD, and there was a significant causal effect of iridocyclitis on psoriasis. Sensitivity analysis further confirmed the reliability and robustness of our MR results. Additionally, we conducted multivariable MR and mediation analysis, demonstrating that the influence of iridocyclitis on the risk of NAFLD was partially mediated by psoriasis.

Previous research has established an association between iridocyclitis and psoriasis 28-31. A study showed that among 101 patients with psoriasis who underwent ophthalmic examinations, 3 out of every 7 patients with psoriatic arthritis had iridocyclitis 32. However, retrospective case-control studies suffer from potential confounding variables and inherent limitations, making it challenging to establish causal relationships based on them. Randomized controlled trials (RCTs) are considered the gold standard for determining causal relationships, but there are currently no RCTs specifically designed to investigate the relationship between iridocyclitis and psoriasis. Existing reports are largely limited to case reports and meta-analyses, thus providing insufficient evidence to establish a clear association or causal relationship between iridocyclitis and psoriasis. Bidirectional MR serves as an effective alternative to overcome the limitations of current research. Therefore, in this study, we conducted univariable MR analysis with iridocyclitis and psoriasis as exposures and outcomes. Our MR analysis revealed a significant causal effect of iridocyclitis on psoriasis, and iridocyclitis a potential risk factor. Previous studies have reported a case of psoriasis and iridocyclitis as early as 1996, suggesting a certain mechanism between the two diseases 29. Subsequent research has found an increased risk of uveitis among psoriasis patients 17. The relationship between psoriasis and non-alcoholic fatty liver disease (NAFLD) has been sparsely researched. However, a recent meta-analysis involving over 10,000 participants from various countries revealed a significant association, indicating that the likelihood of having NAFLD is nearly doubled in patients with psoriasis 33. Therefore, our study results are generally consistent with previous research and indicate a causal relationship between the two diseases. The specific mechanism of this causal relationship remains unclear. However, it is well established that both diseases involve complex interactions between metabolic pathways and the immune system. In this study, the selected instrumental variables were also associated with metabolic disorders. One study indicated that rs112875651 in the tribbles homolog is linked to non-alcoholic fatty liver disease and obesity 34. Additionally, there are reports that rs738408 is widely associated with lipidomic profiles, particularly in phospholipid metabolism 35. Given that both iridocyclitis and psoriasis involve immune system dysregulation and inflammation, we speculate that common immune pathways or genetic factors may contribute to the potential causal association between these diseases. However, further research is needed to elucidate the specific mechanisms by which iridocyclitis as an exposure factor could influence the development or exacerbation of psoriasis.

Our initial MR analysis revealed causal relationship between psoriasis and NAFLD, as well as between iridocyclitis and NAFLD. Previous observational studies have suggested a potential relationship between psoriasis and NAFLD. Researchers included 5,672 adult subjects, among whom 148 had psoriasis and 5,524 did not. The investigation found a higher prevalence of NAFLD among patients with psoriasis, at 32.7%. Psoriasis was associated with NAFLD (odds ratio [OR], 1.67; 95% CI, 1.03-2.70) 36. Our study results are consistent with previous findings and confirm psoriasis as a risk factor for NAFLD. Additionally, our results suggest a causal relationship between iridocyclitis and NAFLD. Although there are currently no observational studies on these two diseases, it is noted that psoriasis patients often have other comorbidities, including iridocyclitis and NAFLD 10, 36. This to some extent supports our study results.

Subsequently, based on the results of univariable MR analysis, we inferred the possible presence of mediation effects among the three diseases and conducted multivariable MR analysis with NAFLD as the outcome and iridocyclitis and psoriasis as exposure factors. The results showed that iridocyclitis significantly influenced the occurrence of NAFLD as a risk factor, while the effect of psoriasis was not significant. Finally, using a multivariable mediation analysis method, we found that iridocyclitis affected the occurrence of NAFLD, with 20.81% of the effect mediated by psoriasis caused by iridocyclitis. Although the exact pathophysiological mechanisms supporting this association remain unclear, an emerging consensus among researchers suggests potential shared etiologies among iridocyclitis, psoriasis, and NAFLD. Proposed explanations for this connection include activated neutrophils in peripheral blood 37, complement system overactivation 38, HLA-B27 39, and metabolic disorders. It is worth noting that our study results differ slightly from previous estimates, as we innovatively propose the existence of mediation effects among the three diseases. This deviation may be due to unavoidable clinical confounding factors in previous studies, which could affect the determination of causal relationships. Additionally, there may be significant overlap among the samples, which could introduce bias. We have assessed sample overlap through the F statistic and calculated the overlap rate, but we will also employ various methods in the future to attempt to eliminate this overlap. MR analysis helps mitigate these issues by integrating genetic instrumental variables. To ensure the robustness and consistency of our causal estimates, we conducted sensitivity and heterogeneity analyses, further strengthening the reliability of our study results. We have also calculated the F statistic and R² values. These metrics can effectively reflect the strength of the association between the instrumental variables and the exposure variables, allowing us to assess whether the instrumental variables are sufficiently strong to capture the variability in the exposure variables.

## Conclusion

Our study demonstrates the existence of univariable and multivariable causal relationships among iridocyclitis, psoriasis, and NAFLD. Our findings emphasize that iridocyclitis is significantly associated with an increased risk of NAFLD, and psoriasis is involved in the mechanism by which iridocyclitis triggers NAFLD, potentially providing future preventive strategies for NAFLD. However, our conclusions are based on genetic susceptibility and do not consider other factors that may lead to the development of iridocyclitis, psoriasis, or NAFLD, such as environmental influences and treatment status. For example, once diagnosed with iridocyclitis, patients undergo immunotherapy, reducing the probability of developing NAFLD or psoriasis. Further investigation is needed to ensure the accuracy and consistency of our results. Continued research on iridocyclitis, psoriasis, and NAFLD is warranted.

## Supplementary Material

Supplementary figures and tables.

## Figures and Tables

**Table 1:** Bidirectional Two-sample Mendelian randomization analysis of the relationship among psoriasis, iridocyclitis, and NAFLD through inverse variance weighted.

Outcome	Exposure	Method	nsnp	b	se	pval	OR
Nonalcoholic fatty liver disease	Iridocyclitis	Inverse variance weighted	10	0.073	0.031	**0.0185**	1.0757
Nonalcoholic fatty liver disease	Psoriasis vulgaris	Inverse variance weighted	9	0.1174	0.0465	**0.0115**	1.1246
Iridocyclitis	Nonalcoholic fatty liver disease	Inverse variance weighted	12	0.0091	0.0281	0.7451	1.0092
Iridocyclitis	Psoriasis vulgaris	Inverse variance weighted	9	-0.0383	0.1155	0.7401	0.9624
Psoriasis vulgaris	Iridocyclitis	Inverse variance weighted	10	0.1595	0.0675	**0.0181**	1.1729
Psoriasis vulgaris	Nonalcoholic fatty liver disease	Inverse variance weighted	12	0.0399	0.0278	0.1519	1.0407

**Table 2:** Heterogeneity test for bidirectional Two-sample MR analysis of the relationship among psoriasis, iridocyclitis, and NAFLD.

Outcome	Exposure	Method	Q	Q_df	Q_pval
Nonalcoholic fatty liver disease	Iridocyclitis	IVW	4.0357	9	0.909
Nonalcoholic fatty liver disease	Psoriasis vulgaris	IVW	4.8658	8	0.7718
Iridocyclitis	Nonalcoholic fatty liver disease	IVW	14.419	11	0.2107
Iridocyclitis	Psoriasis vulgaris	IVW	188.5	8	<0.0001
Psoriasis vulgaris	Iridocyclitis	IVW	127.251	9	<0.0001
Psoriasis vulgaris	Nonalcoholic fatty liver disease	IVW	9.6465	11	0.5624

**Table 3:** Horizontal pleiotropy test for bidirectional Two-sample MR analysis of the relationship among psoriasis, iridocyclitis, and NAFLD through MR-Egger test.

Outcome	Exposure	Egger_intercept	se	pval
Nonalcoholic fatty liver disease	Iridocyclitis	-0.0186	0.0263	0.4994
Nonalcoholic fatty liver disease	Psoriasis vulgaris	0.0011	0.0411	0.9798
Iridocyclitis	Nonalcoholic fatty liver disease	0.006	0.0316	0.8539
Iridocyclitis	Psoriasis vulgaris	0.1901	0.0822	0.054
Psoriasis vulgaris	Iridocyclitis	0.0946	0.0507	0.099
Psoriasis vulgaris	Nonalcoholic fatty liver disease	0.0084	0.0299	0.785

**Table 4:** Results of calculations based on the concentration parameter. The concentration parameter influences the consistency of the mixing distribution and the relative weights of the groups.

Overlap proportion	Bias	Type 1 error rate
0	0	0.05
0.1	0	0.05
0.2	0.001	0.05
0.3	0.001	0.05
0.4	0.002	0.05
0.5	0.002	0.05
0.6	0.003	0.05
0.7	0.003	0.05
0.8	0.004	0.05
0.9	0.004	0.05
1	0.005	0.05

**Table 5:** Results calculated using empirical concentration parameters, typically based on automatically estimated concentration values from sample data.

Overlap proportion	Bias	Type 1 error rate
0	0	0.05
0.1	0	0.05
0.2	0.001	0.05
0.3	0.001	0.05
0.4	0.002	0.05
0.5	0.002	0.05
0.6	0.003	0.05
0.7	0.003	0.05
0.8	0.004	0.05
0.9	0.004	0.05
1	0.005	0.05

**Table 6:** The results of mediation analysis with iridocyclitis as the exposure factor, psoriasis as the mediation factor, and NAFLD as the outcome.

beta1'	beta2	beta3	Mediating effect	Ratio of mediator
0.0713	0.1595	0.1174	0.0187	20.81%

## Abbreviations

MR: Mendelian randomization
NAFLD: non-alcoholic fatty liver disease
IVW: inverse variance weighting
MVMR: multivariable MR
LOO: leave-one-out
NASH: Non-alcoholic Steatohepatitis
SNP: single nucleotide polymorphism
