# Supplementary material for: Correlation Among Psoriasis, Iridocyclitis, and Non-alcoholic Fatty Liver Disease: Insights from Mendelian Randomization and Mediation Analysis
Source: Int J Med Sci. 2025 Jan 1;22(1):121–31. doi: 10.7150/ijms.102369 (PMC11659831; doi:10.7150/ijms.102369)
Supplement: Supplementary file 1 — Supplementary figures and tables. [file ijmsv22p0121s1.zip › supplementary.pdf]

Supplementary materials

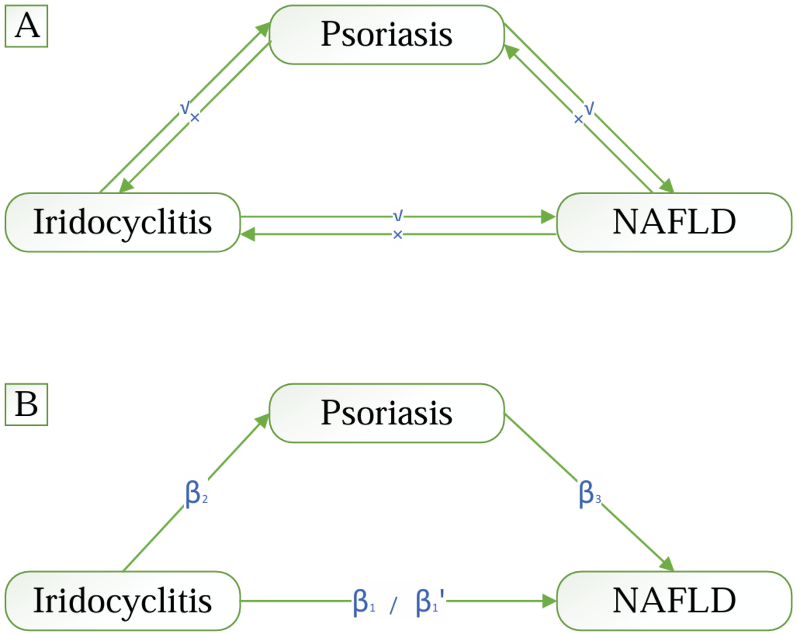

Supplementary Figure 1| Mediation effect analysis. (A) Overview of the causal relationships among psoriasis, iridocyclitis, and NAFLD. (B) Orienting of the mediation effect analysis.

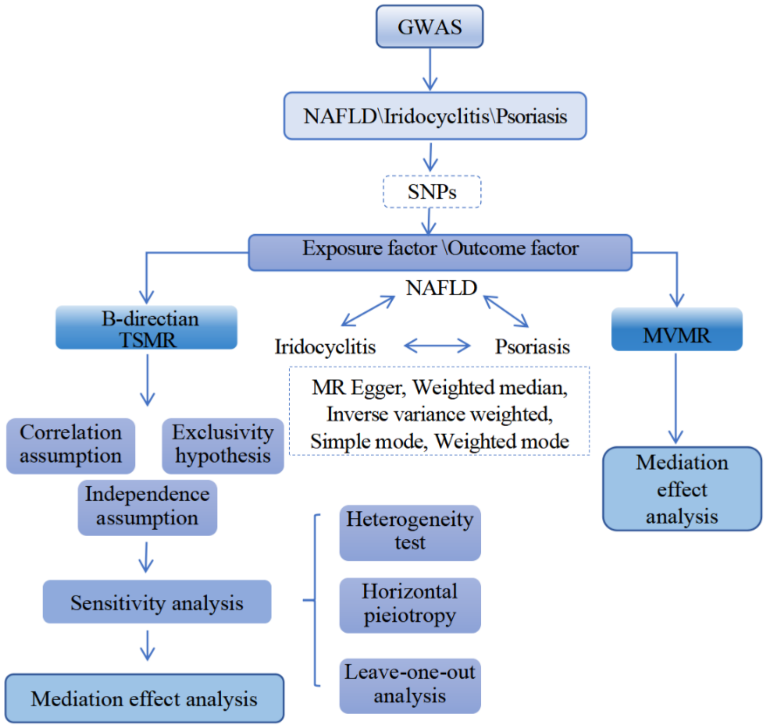

Supplementary Figure 2| Experimental flow chart

outcome	exposure	method	nsnp	b	se	pval	OR
Nonalcoholic fatty liver disease	Iridocyclitis	MR Egger	10	0.0916	0.0407	0.0543	1.0959
Nonalcoholic fatty liver disease	Iridocyclitis	Weighted median	10	0.0759	0.0351	0.0308	1.0788
Nonalcoholic fatty liver disease	Iridocyclitis	Inverse variance weighted	10	0.073	0.031	0.0185	1.0757
Nonalcoholic fatty liver disease	Iridocyclitis	Simple mode	10	0.0517	0.0634	0.4356	1.0531
Nonalcoholic fatty liver disease	Iridocyclitis	Weighted mode	10	0.0745	0.0342	0.0575	1.0774
Nonalcoholic fatty liver disease	Psoriasis vulgaris	MR Egger	9	0.1157	0.0803	0.1929	1.1226
Nonalcoholic fatty liver disease	Psoriasis vulgaris	Weighted median	9	0.125	0.0557	0.0248	1.1332
Nonalcoholic fatty liver disease	Psoriasis vulgaris	Inverse variance weighted	9	0.1174	0.0465	0.0115	1.1246
Nonalcoholic fatty liver disease	Psoriasis vulgaris	Simple mode	9	0.1612	0.0919	0.1176	1.1749
Nonalcoholic fatty liver disease	Psoriasis vulgaris	Weighted mode	9	0.1198	0.0552	0.0619	1.1272
Iridocyclitis	Nonalcoholic fatty liver disease	MR Egger	12	-0.0015	0.0634	0.9817	0.9985
Iridocyclitis	Nonalcoholic fatty liver disease	Weighted median	12	0.0066	0.035	0.8497	1.0066
Iridocyclitis	Nonalcoholic fatty liver disease	Inverse variance weighted	12	0.0091	0.0281	0.7451	1.0092
Iridocyclitis	Nonalcoholic fatty liver disease	Simple mode	12	0.0094	0.0556	0.8692	1.0094
Iridocyclitis	Nonalcoholic fatty liver disease	Weighted mode	12	0.0094	0.043	0.8314	1.0094
Iridocyclitis	Psoriasis vulgaris	MR Egger	9	-0.3412	0.1606	0.0713	0.7109
Iridocyclitis	Psoriasis vulgaris	Weighted median	9	-0.1636	0.0295	0	0.8491
Iridocyclitis	Psoriasis vulgaris	Inverse variance weighted	9	-0.0383	0.1155	0.7401	0.9624
Iridocyclitis	Psoriasis vulgaris	Simple mode	9	-0.0331	0.0707	0.6519	0.9674
Iridocyclitis	Psoriasis vulgaris	Weighted mode	9	-0.1501	0.0284	0.0007	0.8606
Psoriasis vulgaris	Iridocyclitis	MR Egger	10	0.0646	0.0785	0.4342	1.0667
Psoriasis vulgaris	Iridocyclitis	Weighted median	10	0.1137	0.0212	0	1.1205
Psoriasis vulgaris	Iridocyclitis	Inverse variance weighted	10	0.1595	0.0675	0.0181	1.1729
Psoriasis vulgaris	Iridocyclitis	Simple mode	10	0.3999	0.1363	0.0167	1.4916
Psoriasis vulgaris	Iridocyclitis	Weighted mode	10	0.1208	0.019	0.0001	1.1284
Psoriasis vulgaris	Nonalcoholic fatty liver disease	MR Egger	12	0.025	0.06	0.6855	1.0253
Psoriasis vulgaris	Nonalcoholic fatty liver disease	Weighted median	12	0.0781	0.0388	0.0438	1.0812
Psoriasis vulgaris	Nonalcoholic fatty liver disease	Inverse variance weighted	12	0.0399	0.0278	0.1519	1.0407

Psoriasis vulgaris	Nonalcoholic fatty liver disease	Simple mode	12	0.0858	0.0639	0.2063	1.0896
Psoriasis vulgaris	Nonalcoholic fatty liver disease	Weighted mode	12	0.0947	0.0481	0.0749	1.0994

Supplementary Table 1| The UVMR results of five different methods, including MR Egger, weighted median, inverse variance weighted, simple mode, weighted mode.

Supplementary Table 2 F-value of SNP: psoriasis-iridocyclitis.

Supplementary Table 3: F-value of SNP: Psoriasis - Nonalcoholic Fatty Liver Disease.

Supplementary Table 4: F-value of SNP: Iridocyclitis-Psoriasis.

Supplementary Table 5: F-value of SNP: Non-alcoholic fatty liver disease - psoriasis.

Supplementary Table 6: F-value of SNP: Non-alcoholic fatty liver disease - psoriasis.

Supplementary Table 7: F-value of SNP: Non-alcoholic fatty liver disease - iridocyclitis.
