# Supplementary figures and images for: Correlation Among Psoriasis, Iridocyclitis, and Non-alcoholic Fatty Liver Disease: Insights from Mendelian Randomization and Mediation Analysis
Source: Int J Med Sci. 2025 Jan 1;22(1):121–31. doi: 10.7150/ijms.102369 (PMC11659831; doi:10.7150/ijms.102369)

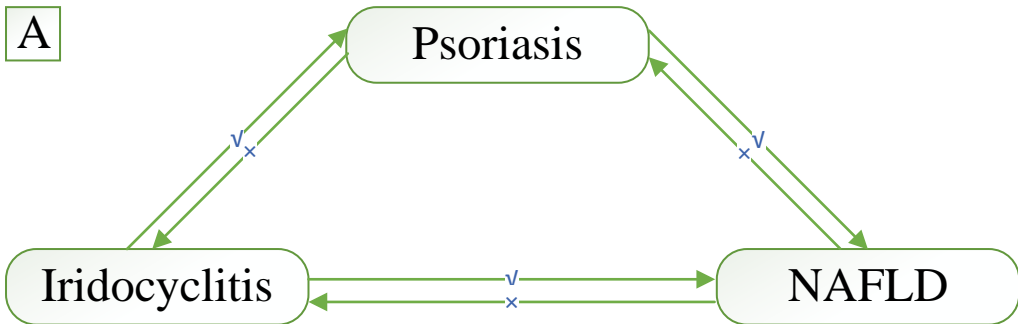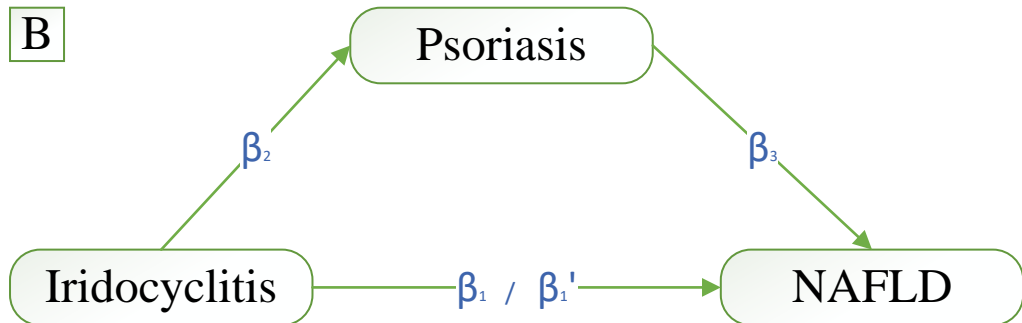

Supplement: Supplementary file 1 — Supplementary figures and tables. [file ijmsv22p0121s1.zip › Supplementary Figure/Supplementary Fig1.pdf]

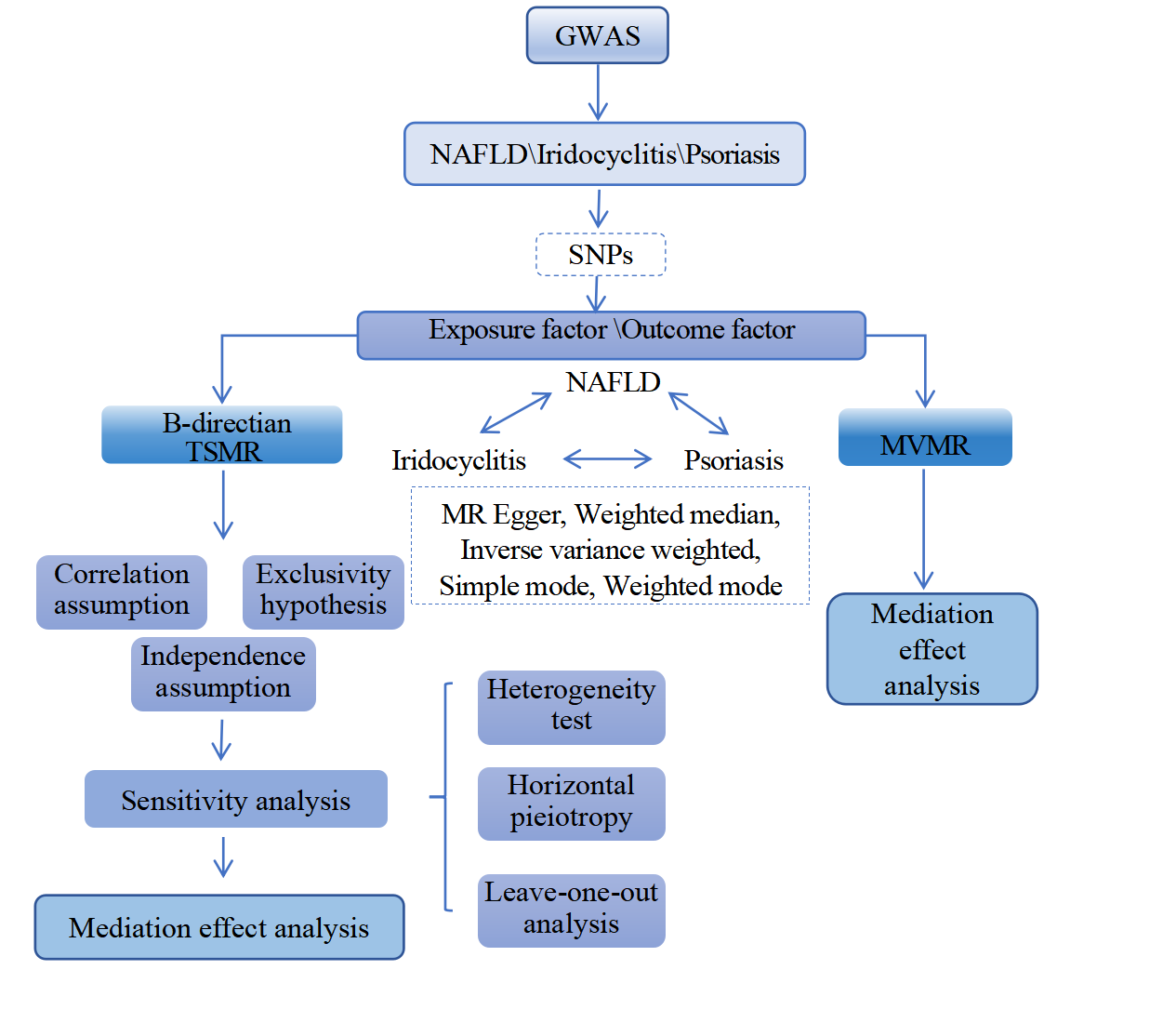

Supplement: Supplementary file 1 — Supplementary figures and tables. [file ijmsv22p0121s1.zip › Supplementary Figure/Supplementary Figure 2 .tif]
